# Ultrashort wave therapy promotes traumatic brain injury recovery by suppressing neuroinflammation

**DOI:** 10.4103/NRR.NRR-D-24-01479

**Published:** 2025-05-06

**Authors:** Chuang Xu, Jinwei Liu, Qiaozhen Qin, Heyang Zhang, Xiaotong Li, Yue Chen, Zhenhua Xu, Fang Wang, Nihui Zhang, Zhen Zhang, Yifei Tan, Lingyu Zhang, Guilin Chen, Liu Liu, Weiwei Xing, Yan Wang, Huaqiang Ruan, Xiaoxia Jiang, Nan Peng

**Affiliations:** 1 Graduate School of the Chinese PLA General Hospital, Beijing, China; 2 Beijing Institute of Basic Medical Sciences, Beijing, China; 3 Department of Rehabilitation Medicine, The Second Medical Center & National Clinical Research Center for Geriatric Diseases, Chinese PLA General Hospital, Beijing, China; 4 Anhui Medical University, Hefei, Anhui Province, China

**Keywords:** astrocyte, cognitive disorders, emotional disorders, lesion repair, neuroinflammation, neurological disorders, Piezo1, traumatic brain injury, ultrashort wave

## Abstract

Despite growing treatments for traumatic brain injury, there is still no ideal strategy for efficiently mitigating these processes. Ultrashort wave therapy, a type of physical factor therapy, has been widely used in various clinical treatments. However, its effects on traumatic brain injury and the underlying mechanisms are not well understood. In this study, we demonstrate that ultrashort wave treatment can significantly promote injury repair and alleviate emotional and cognitive disorders. Our data showed that ultrashort wave reduced the levels of pro-inflammatory factors and inhibited neuroinflammation. *In vitro* experiments showed that ultrashort wave inhibited activation of C8-D1A astrocytes and BV2 microglia. Furthermore, traumatic brain injury induced the expression of Piezo1, while ultrashort wave effectively suppressed this high expression. Administration of Yoda1, a Piezo1 agonist, to traumatic brain injury mice reversed the beneficial effects of ultrashort wave. Consistently, Yoda1 also reversed the inhibitory effect of ultrashort wave on activation of C8-D1A astrocytes. These findings indicate that ultrashort wave is an ideal therapeutic strategy for traumatic brain injury, which works by inhibiting Piezo1, reducing neuroinflammation, and promoting nerve repair after traumatic brain injury.

## Graphical abstract

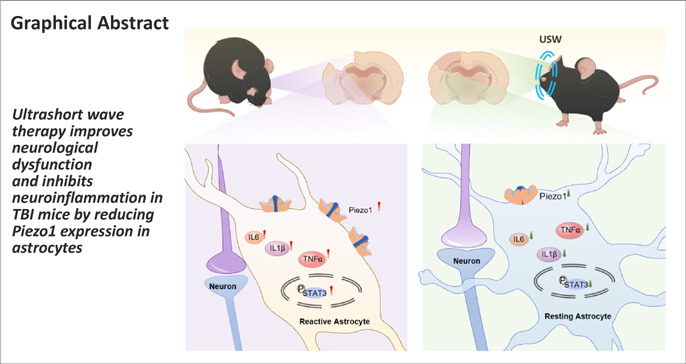

## Introduction

Traumatic brain injury (TBI) constitutes a primary cause of death and long-term disability (Izzy et al., 2023). TBI causes neural tissue damage, neuroinflammation, and nerve dysfunction, which is associated with anxiety, depression, and motor and cognitive impairments (Panza et al., 2022). Moreover, evidence suggests that neuroinflammation persists for a long time after TBI, inducing a series of neurodegenerative diseases such as Alzheimer’s disease, major depressive disorder, and Parkinson’s disease (van Vliet et al., 2020). Due to the complex mechanism of secondary brain injury (including neuroinflammation, brain edema, blood–brain barrier disruption, release of excitatory neurotransmitters, and oxidative stress), there remains no widely recognized treatment for TBI and current options are limited to supportive care (Yang et al., 2022b).

TBI can trigger a series of inflammatory responses, activate microglia and astrocytes, result in apoptosis and death of nerve cells, and eventually aggravate brain injury (Lagraoui et al., 2017; Jacquens et al., 2024). The current clinical management of TBI is mainly symptomatic, with high-dose steroid hormones potentially increasing the risk of mortality (Yang et al., 2022b). Some novel treatments based on preclinical research have good curative effects; this includes low-dose ionizing radiation (Au et al., 2024), intravenous immunoglobulin (Willis et al., 2023), intranasally administered human mesenchymal stem cell-derived extracellular vesicles (Kodali et al., 2023), stem cells, and gene therapy (including DNA, RNA, and microRNA; Jin et al., 2025). However, these treatments need extensive clinical trials to refine and optimize the treatment parameters and safety (Xue et al., 2020; Jones et al., 2023; Soltani et al., 2023). Therefore, identifying a safe and efficacious non-drug treatment method is urgently needed.

Ultrashort wave (USW) therapy is a physical factor therapy that elicits biological effects through electromagnetic oscillation effects (Aydin and Akar, 2011; Bouji et al., 2020; Tenenbaum, 2024). To achieve optimal tissue penetration, instruments for medical USW therapy typically operate at specific frequencies (e.g., 40.68 or 27.12 MHz). Influenced by the rapidly changing electric field, high-frequency USW therapy stimulates rapid oscillations and movements of ions and charged particles within the body, (Aydin and Akar, 2011; Bouji et al., 2020; Mumtaz et al., 2022). Due to its potent anti-inflammatory and tissue repair effects, USW has been widely used in the clinical treatment of infectious diseases, including tympanitis, nasosinusitis, and erysipelas (Guo et al., 2021; Huang et al., 2021). Research on spinal cord injury found that USW can lower the levels of pro-inflammatory factors and inhibit activation of astrocytes and microglia, indicating its potential in inhibiting inflammation and promoting functional recovery (Na et al., 2020; Wang et al., 2021). Our previous work applying USW combined with platelet-rich plasma to rabbit sciatic nerve compression injury demonstrated that USW can promote the formation of new blood vessels and accelerate the growth of myelin and axons (Zhu et al., 2020). Given these promising findings, we speculate that USW maybe a beneficial therapeutic approach for TBI.

In this study, we determined whether USW therapy can effectively inhibit neuroinflammation and facilitate neural repairment following TBI. We also sought to identify the potential mechanisms underlying these therapeutic effects.

## Methods

### Animals and study design

To avoid the potential confounding effects of fluctuating estrogen levels and estrous cycles in female mice, male mice were used in all experiments. A total of 120 male C57BL/6N mice aged 8–10 weeks were purchased from Beijing Vital River Laboratory Animal Technology (Beijing, China, license No. SCXK (Jing) 2021-0006). Their body weight was approximately 22–24 g. All mice were housed in a standard animal facility under conditions of a controlled temperature (22–26°C) and a 12/12-hour light/dark cycle with free access to water and food. All animal experiments were carried out in accordance with the Guidelines for the Care and Use of Experimental Animals approved by Beijing Institute of Basic Medical Sciences. The Ethical Review Committee of Beijing Institute of Basic Medical Sciences approved all experimental protocols (approval No. IACUC-DWZX-2021-759) on November 16, 2021. All experiments were designed and reported according to the Animal Research: Reporting of *In Vivo* Experiments (ARRIVE) guidelines (Percie du Sert et al., 2020). All surgery was performed under 2,2,2-tribromoethanol anesthesia, and all efforts were made to minimize suffering.

To determine the therapeutic effect of USW on TBI mice, the animals were divided into Sham, TBI, and TBI + USW groups using a random number table method (Qu et al., 2023); each group had 16 mice. The results were verified by behavioral, histological, and molecular experiments.

To confirm that USW improves nerve damage after TBI by inhibiting Piezo1 protein, mice were randomly divided into five groups: Sham group, TBI group, TBI + USW group, TBI with Piezo1 inhibitor group (TBI + Dooku1), and USW with Piezo1 agonist group (TBI + USW + Yoda1); each group had 12 mice. Mice were intraperitoneally injected with Dooku1 (10 mg/kg [Qu et al., 2023]; Abmole, Houston, TX, USA, M10812) and Yoda1 (1 mg/kg [Matrongolo et al., 2023]; Abmole, M9372) at 0.5 hours after brain injury, and then injected at a fixed time every day until day 7. Behavioral experiments were then performed.

### Construction of traumatic brain injury models

Animals were subjected to TBI using a controlled cortical impact injury model (Qin et al., 2022). Before controlled cortical impact injury, each animal was intraperitoneally injected with 2,2,2-tribromoethanol (350 mg/kg, Sigma–Aldrich, Burlington, MA, USA, T48402) for anesthesia. Animals were placed on a stereotaxic frame attached to a temperature-controlled heating pad (37°C) with their scalp depilated. After exposing the skull, a 4-mm craniectomy was performed over the cortex (3.0 mm anterior–posterior [AP] and 2.0 mm medial–lateral [ML] to bregma). A pneumatically operated metal impactor (Zhongshi Dichuang Tech, Beijing, China, diameter = 3 mm) impacted the brain at a velocity of 3.5 m/s, reaching a depth of 1.5 mm below the dura mater layer. The impactor was held in the brain for 0.4 seconds. Sham mice underwent all these procedures, except controlled cortical impact.

### Ultrashort wave treatment

**Figure 1:**
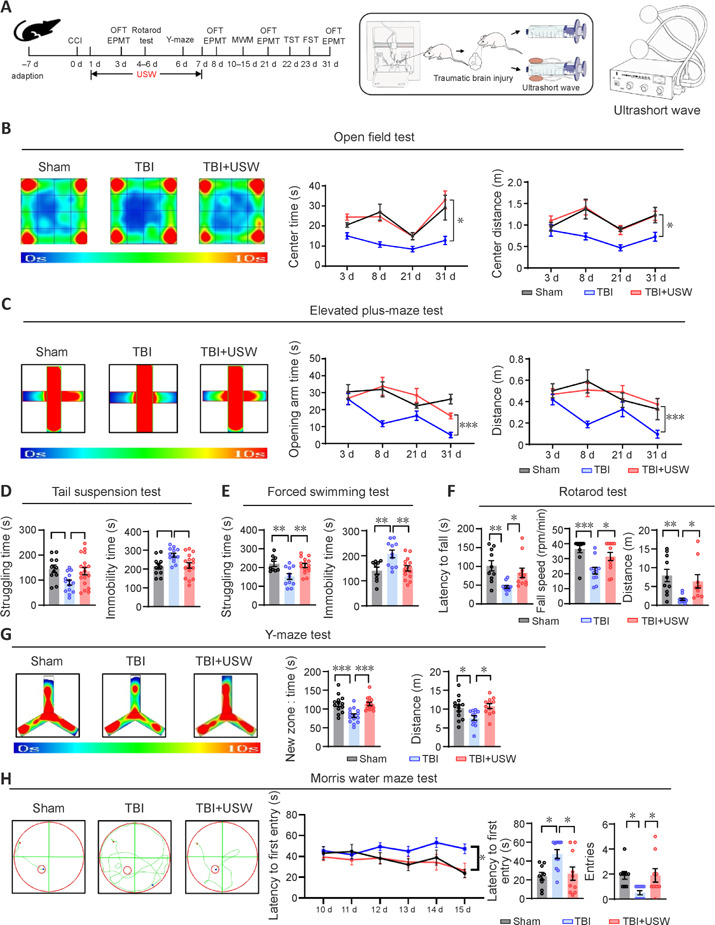
USW improves anxiety, depression, and motor and cognitive deficits in mice with TBI. (A) Schematic of the experimental timeline and USW equipment. (B, C) USW caused markedly lower anxiety levels in TBI mice, measured by the open field test (B) and elevated plus maze test (C). (D, E) USW resulted in lower levels of depression in TBI mice, measured by the tail suspension test (D) and forced swimming test (E). (F) USW caused higher levels of motor ability in TBI mice, measured by the rotarod test. (G, H) USW resulted in a higher level of cognitive ability in TBI mice, measured by the Y-maze test (G) and Morris water maze test (H). Data are expressed as mean ± SEM (*n* = 14 [Sham] or 16 [TBI and TBI + USW]). *P < 0.05, **P < 0.01, ***P < 0.001 (one-way analysis of variance followed by Dunnett’s *post hoc* test). CCI: Controlled cortical impact; EPMT: elevated plus-maze test; MWM: Morris water maze test; ns: no significant; OFT: open field test; TBI: traumatic brain injury; TST: tail suspension test; USW: ultrashort wave.

A five-sense USW therapy instrument was used (Shantou Medical Equipment Factory Co., Ltd, Shaotou, China, DL-CII) with a frequency of 40.68 MHz. Prior to use, the machine was preheated for 5 minutes. During treatment intervention, two 4-cm diameter electrodes were placed parallel to each other on both sides of the brain, approximately 2 cm from the scalp. The machine was set at level 1 with no heat output. One day (24 hours) after TBI, mice in the TBI + USW and TBI + USW + Yoda1 groups were treated with USW once a day for 7 minutes each time for 7 consecutive days. Mice in the Sham and TBI groups were also placed between the electrodes of the machine, but the machine was not turned on. As shown in **Figure 1A**, the mouse was fixed in a 50 mL syringe tube, and the bottom of the syringe was pushed until the head could not move freely. The syringe tube was cut into strips to ensure that the mouse could breathe freely (Na et al., 2020; Wang et al., 2021).

### Behavioral tests

#### Open field test

In the open field test, anxiety is reflected by the amount of time rodents spend at the edges of the box, avoiding the center of the open field (Giannoccaro et al., 2019). Total distance travelled, entries within each zone, and time spent and distance traveled within each zone were recorded. To test for exploration of the center, data was analyzed over a 5-minute period.

#### Elevated plus maze test

Anxiety-like behavior was examined using the elevated plus maze (Hedges et al., 2021). The experimental equipment (Zhongshi Dichuang Tech) is shaped like a “plus sign,” consisting of two open arms and two closed arms. The equipment is placed 100 cm above the ground to create an exposed and dangerous environment. Its principle is based on the instinctive behavior of rodents. Specifically, rodents have an innate tendency to explore new environments, but they also have a fear of open and elevated spaces. Briefly, mice were placed in the center area facing an open arm and allowed to explore freely over a 5 minute period. Anxiety-like behavior was quantified by time spent in the open arms across the testing period.

#### Rotarod test

The rotarod test was performed to assess motor coordination and balance ability (Smith et al., 2020). The experimental equipment (Zhongshi Dichuang Tech) rotates the rotating rod through a driving system. During the experiment, mice are placed on the rotating rod. To avoid falling, mice will instinctively walk or run on the rotating rod. As the experiment progresses, the animals will gradually get fatigued and eventually fall off the rotating rod. First, mice were trained for 2 days. On the first training day, the speed was set to 5 r/min and mice were given 5 minutes on the rotarod. On the second training day, the speed was set to 40 r/min and the time was 5 minutes. The mice were trained four times a day, and the interval between each training session was at least 30 minutes. Then on the third test day, the speed was set at 40 r/min with an upper time of 5 minutes. The latency to fall from the rotarod and the distance and speed of the rod when the mice fell was quantified.

#### Beam balance test

The beam balance test was used to assess motor coordination and balance ability (Tsai et al., 2019). The experimental equipment (Zhongshi Dichuang Tech) consists of a beam and dark box (target platform). The beam is 120 cm long, 2.1 cm wide, and was situated at a height of 80 cm above the ground. Before the experiment, the mice were first allowed to acclimatize to the experimental environment (beam and dark box). Mice were placed 100 cm away from the dark box. The time taken for the mouse to arrive at the box was recorded. Mice were then rested in the box for 1 minute. Mice were trained for 2 days, with three training sessions each day. Then the actual experiment was performed.

#### Y-maze test

Spatial memory was examined using the Y-maze (Fronza et al., 2022). The Y-maze (Zhongshi Dichuang Tech) consists of three arms of the same length and width. The three arms meet at the center at an angle of approximately 120°, and the overall shape resembles the letter “Y.” The Y-maze experiment is mainly based on the animals’ desire to explore new environments and can be used to evaluate spatial cognitive abilities. One closed arm of the apparatus was initially designated as the novel arm. The mouse was then placed into the device and allowed to explore freely for 15 minutes. This initial exploration period was to acclimate the mouse to the general environment of the apparatus with the novel arm remaining inaccessible. After 30 minutes, the novel arm was opened, and the mouse was reintroduced to the device for a second exploration session lasting 5 minutes. During this session, the time and distance spent exploring the novel arm were recorded. Data on exploration time and distance in the novel arm was used to determine spatial memory.

#### Morris water maze test

The morris water maze (MWM) was used to assess spatial learning and memory (Xu et al., 2020). The experimental device (Zhongshi Dichuang Tech) was composed of a circular pool (150 cm in diameter and 50 cm in height, divided into four quadrants), with a platform, camera system, and an animal behavior trajectory analysis system. The circular platform (10 cm × 10 cm, 1 cm from the water level) was hidden in the middle of the fourth quadrant. The pool was dyed white with non-toxic titanium dioxide transparent agent, and the water temperature is maintained at 21–25°C. The 10^th^–14^th^ days were the training period. During each experiment, the mice were placed facing the pool wall into the pool from each of the four quadrants. Due to their survival instinct, mice swim in the pool until they find the hidden platform under the water surface. Each training time is 60 seconds. If the survival platform is found, the recording time is 60 seconds. If the animal is guided to find the platform, the recording time is 60 seconds. Mice were allowed to stay on the platform for 10 seconds. During the test period, the escape platform was removed, and the number of times the mice crossed the escape platform and the time of first platform crossing were recorded.

#### Tail suspension test

The tail suspension test was used to examine depression-like behavior (Zhang et al., 2022). The experimental equipment (Zhongshi Dichuang Tech) consists of a suspension device and camera device. The principle of the experiment is based on the fact that animals will initially struggle to get out of an inescapable predicament. However, after a period of attempts, they enter a motionless state. This motionless state is considered to reflect a “despair” emotion, which is similar to the depressive state in humans. By observing and recording behavioral indicators such as immobility time, the degree of depressive-like behavior can be evaluated. The mouse was suspended upside down by its tail for 5 minutes in a box approximately 10 cm from the ground. This ensured that the mouse could not touch the ground. The time it took for the mouse to stop struggling and remain motionless was recorded.

#### Forced swimming test

The forced swimming test was performed to assess depression-like behavior (Zhang et al., 2024). The experimental equipment (Zhongshi Dichuang Tech) consists of a swimming container (diameter of 20 cm and height of 25 cm) and camera device. The theoretical basis of this experiment is based upon the fact that in ‘inescapable water,’ mice will initially struggle desperately to escape their predicament. However, when they cannot escape, they enter a motionless state, only making the necessary movements to keep their heads above water. This motionless state is regarded as “behavioral despair,” which is similar to the sense of helplessness and despair in human depression. By measuring indicators such as immobility time, the degree of their depressive-like behavior can be evaluated. Clean tap water was used. The water temperature was controlled between 23–25°C, and the water level was deep enough to ensure that the animals could not touch the bottom of the container with their limbs or tails. The experiment lasted for 5 minutes and the time taken to produce a motionless state was recorded.

### Tissue collection

Mice were anesthetized by intraperitoneal injection of 2,2,2-tribromoethanol (350 mg/kg, Sigma–Aldrich). The chest was opened using surgical instruments and the ribs freed to fully expose the heart. The right atrium was cut after inserting a disposable blood sampling needle into the apex of the heart. Mice were perfused with normal saline until the liver was white and the outflow fluid was clear. Then 4% paraformaldehyde was used to fix the brain tissue. Tremble and convulsion of the limbs and tail were regarded as successful fixation. Tissues used for real-time polymerase chain reaction (PCR) and western blotting were not fixed.

### Cell culture

The astrocyte line C8-D1A (Cat# CL-0506, RRID: CVCL_6379) and microglia line BV2 (Cat# CL-0493, RRID:CVCL_0182) were purchased from Keygen Biotech (Taizhou, Jiangsu, China). Cells were incubated at 37°C in 5% CO_2_ with cell complete medium (Dulbecco’s modified Eagle medium high glucose, 10% fetal bovine serum, and 1% penicillin/streptomycin). For USW intervention, the same equipment was used as described for the animal experiments. The treatment parameters were also consistent. For USW intervention, the cell culture dish was briefly sealed with parafilm and a 2-cm plastic pad placed at the bottom of the dish to ensure that neither electrode touched the bottom. Sham intervention was treated in the same way, but the equipment was not turned on.

To determine the effect of USW on neuroinflammation, C8-D1A and BV2 cells were divided into: Control group, tumor necrosis factor-alpha (TNF-α) + interleukin (IL)-1β group, and TNF-α + IL-1β + USW group. For inflammatory activation, cells in the TNF-α + IL-1β and TNF-α + IL-1β + USW groups were first induced with 120 ng/mL TNF-α (PeproTech, Rocky Hill, NJ, USA, 315-01A) and 40 ng/mL IL-1β (PeproTech, 211-11B). After 12 hours, cells in the TNF-α + IL-1β + USW group were subjected to one session of USW. Cells in the Control and TNF-α + IL-1β groups were also placed between the electrodes of the machine without turning it on. Briefly, the cell culture dish was placed on a plastic pad 2 cm in height to ensure that neither electrode touched the bottom of the dish. After 24 hours, the cells were collected for immunofluorescence staining, real-time PCR, and western blotting experiments.

To confirm that USW inhibits neuroinflammation by reducing Piezo1 protein, C8-D1A cells were randomly divided into five groups: Control group, TNF-α + IL-1β group, TNF-α + IL-1β + USW group, TNF-α + IL-1β + GsMTx4 group, and TNF-α + IL-1β + USW + Yoda1 group. Inflammatory activation of C8-D1A cells was stimulated by TNF-α and IL-1β. The Piezo1 agonist, Yoda1 (10 µM [Velasco-Estevez et al., 2020]; Abmole, M9372) and inhibitor GsMTx4 (0.5 µM [Velasco-Estevez et al., 2020]; Abmole, M10039) were added at the same time. After 12 hours, cells were subjected to one session of USW, and samples collected 24 hours later for real-time PCR and western blotting experiments.

### Immunofluorescence staining

Brain samples were dehydrated and sectioned. Sections were fixed with 4% paraformaldehyde for 1 hour. After, samples were blocked with 0.1% Triton/serum blocking solution at room temperature (20–25°C) for 1 hour. Next, diluted primary antibody (1:200) was added and incubated overnight at 4°C. The corresponding secondary antibodies were added and incubated at room temperature for 1 hour before washing with phosphate buffered saline more than three times. Sections were stained with 4′,6-diamidino-2–phenylindole (Thermo Fisher Scientific, Waltham, MA, USA, D1306). Images were observed under a fluorescence microscope (Olympus Corporation, Beijing, China, CKX53). The primary antibodies used were: glial fibrillary acidic protein (GFAP, mouse, EMD Millipore, Billerica, MA, USA, Cat# 3380386, RRID: AB_2314536), ionized calcium-binding adapter molecule 1 (Iba1, rabbit, Abcam, Cambridge, UK, Cat# ab178847, RRID: AB_2832244), microtubule-associated protein 2 (MAP2, rabbit, Abcam, Cat# ab32454, RRID: AB_776174), doublecortin (DCX, rabbit, Cell Signaling Technology, Danvers, MA, USA, Cat# 91954, RRID: AB_10640853), and Piezo1 (rabbit, Abcam, Cat# ab128245, RRID: AB_2365717). The secondary antibodies were Alexa Fluor 488 (mouse, 1:200, Jackson ImmunoResearch, West Grove, PA, USA, Cat# 715-546-150, RRID: AB_3595484) and Cy3 (rabbit, 1:200, Jackson ImmunoResearch, Cat# 711-165-152, RRID: AB_10893626). Mean fluorescence intensity was analyzed using ImageJ 1.54f software (National Institutes of Health, Bethesda, MD, USA) (Schneider et al., 2012).

### Hematoxylin–eosin staining

Brain, heart, liver, spleen, lung, and kidney tissue were dissected, fixed in 4% paraformaldehyde overnight, embedded in paraffin at 4°C, and cut into thin slices (5 µm thickness). Paraffin-embedded sections were subjected to staining using Harris hematoxylin (Abcam, Cat# ab245880) for 3–8 minutes. They were then subsequently stained with eosin for 1–3 minutes to facilitate microscopic examination.

### Routine blood indices and blood biochemical analysis

Approximately 1 mL of peripheral blood was extracted from mice. For the detection of routine blood indices, peripheral blood was added into ethylenediaminetetraacetic acid anticoagulant tubes, according to the manufacturer’s protocol (ServiceBio Inc., Wuhan, China). A three-part differential hematology analyzer (Mindray, Shenzhen, China) was used to detect blood cells. For blood biochemical analysis, peripheral blood was left to stand for 1 hour at 20–25°C and then centrifuged for 10 minutes (4°C, 3000 r/min). A biochemical analyzer (Rayto, Shenzhen, China) was used to conduct blood biochemical analysis.

### Real-time polymerase chain reaction

Lesioned brain tissue and cells were immersed in Trizol (Invitrogen, Cat# 10,296,010). Total RNA was prepared by chloroform extraction and isopropanol precipitation according to the manufacturer’s recommendations (Ziller et al., 2015). Total RNA was converted to complementary DNA (cDNA) using 5× RT Master Mix (Toyobo, Osaka, Japan, Cat# 037,400). Real-time PCR was performed with 2× T5 Rapid qPCR mixture (SYBR) (Tsingke, Cat# TSE 202). Relative gene expression was calculated using the 2^–ΔΔCt^ method (Qin et al., 2022) with normalization to β-actin. Primer sequences are shown in **Additional Table 1**.

**Additional Table 1:** Sequences of primers used for real-time polymerase chain reaction analysis

Name	Primer sequence (5’-3’)
*β-Actin*	F: TTCTTTGCAGCTCCTTCGTTGCCG R: TGGATGGCTACGTACATGGCTGGG
*GDNF*	F: AAAGACTGAAAAGGTCACCAGA R: CAAACCCAAGTCAGTGACATTT
*NGF*	F: TCAGACACTCTGGATCTAGACT R: CTGTTGTTAATGTTCACCTCGG
*BDNF*	F: CCCATGAAAGAAGTAAACGTCC R: CCTTATGGTTTTCTTCGTTGGG
*TNF-α*	F: GGACTAGCCAGGAGGGAGAACAG R: CCAGTGAGTGAAAGGGACAGAAC
*IL-1β*	F: TCGCAGCAGCACATCAACAAGAG R: AGGTCCACGGGAAAGACACAGG
*IL-6*	F: TCTGGAGCCCACCAAGAACGATAG R: GTCACCAGCATCAGTCCCAAGAAG
*Cx3cr1*	F: AGCTCACGACTGCCTTCTTC R: GTCCGGTTGTTCATGGAGTT
*CD68*	F: GAAATGTCACAGTTCACACCAG R: GGATCTTGGACTAGTAGCAGTG
*Aif-1*	F: CCGAGGAGACGTTCAGCTAC R: GACCAGTTGGCCTCTTGTGT
*GBP2*	F: TCAGCAGCACCTTCATCTACAACAG R: AGCCCACAAAGTTAGCGGAATCG
*H2-D1*	F: AGAGCAGTGTTCCGAGTGAGCCTGA R: CCCTGTGAGCTTGGGTTCAGTGCCG
*Piezo 1*	F: CCTGTTACGCTTCAATGCTCT R: GTGTAGGCATATCTGAAAGGCAA
*Piezo2*	F: CTCGGCTGTGTACTTCTTTGT R: AGCAATGGGTCGAAAGTTCGG
*TRPV1*	F: COGGCTTTITGGGAAGCGT R: GAGACAGGTAGGTCATCCAC
*TRPV4*	F: AAACCTGCGTATCAAGTTCCAG R: CCGTAGTCGAACAAGCAATCCA
*TRAAK*	F: CACTGGTGCTGCTTTACTTGG R: TGGTCCCTCAGAAACTGGTC

Aif-1: allograft inflammatory factor-1; BDNF: brain-derived neurotrophic factor; Cx3cr1: C-X3-C motif chemokine receptor 1; F: forward; GBP2: guanylate-binding protein 2; GDNF: glial cell-derived neurotrophic factor; H2-D1: histocompatibility 2, D region locus 1; IL: interleukin; NGF: nerve growth factor; R: revese; TNF-α: tumor necrosis factor-alpha; TRAAK: potassium channel subfamily K member 4; TRPV1 : transient receptor potential vanilloid 1; TRPV4: Transient receptor potential vanilloid 4.

### Western blot assay

Western blotting was carried out as previously described (Zhang et al., 2022). Lesioned brain tissue and cells were lysed using radioimmunoprecipitation assay buffer to extract protein. Protein concentration was determined using a BCA protein assay kit (Thermo Fisher Scientific, Cat# 23225). Whole protein fractions of samples were subsequently separated by sodium dodecyl sulfate–polyacrylamide gel electrophoresis using 12% Tris gels and blotted onto polyvinylidene fluoride membranes. Membranes were incubated with the appropriate primary antibodies overnight at 4°C. After incubation with secondary antibodies, labeled proteins were visualized using an ECL chemiluminescence detection kit (Merck Millipore, Billerica, MA, USA, Cat# 34075). The following primary antibodies were used: anti-glyceraldehyde-3-phosphate dehydrogenase (GAPDH; rabbit, 1:1000, ABclonal, Wuhan, China, Cat# AC001, RRID: AB_2619673), anti-β-actin (rabbit, 1:1000, ABclonal, Cat# AC004, RRID: AB_2769856), anti-signal transducer and activator of transcription 3 (STAT3, mouse, 1:1000, Cell Signaling Technology, Cat# 14047, RRID: AB_2728821), anti-phosphorylated signal transducer and activator of transcription 3 (p-STAT3, mouse, 1:1000, Cell Signaling Technology, Cat# 4093, RRID: AB_2255568), anti-Piezo1 (rabbit, 1:1000, Abcam, Cat# ab128245, RRID: AB_2365717), anti-GFAP (mouse, 1:1000, EMD Millipore, Cat# AB5541, RRID: AB_1150393), anti-caspase-3 (rabbit, 1:1000, Cell Signaling Technology, Cat# 9662, RRID: AB_2758506), anti-guanylate-binding protein 2 (GBP2; rabbit, 1:1000, Proteintech, Wuhan, China, Cat# 11854-1-AP, RRID: AB_2109336), anti-brain-derived neurotrophic factor (BDNF; rabbit, 1:1000, Proteintech, Cat# 25699-1-AP, RRID: AB_3669493), anti-TNF-α (rabbit, 1:1000, Proteintech, Cat #26162-1-AP, RRID: AB_3085845), anti-CD68 (rabbit, 1:1000, Abcam, Cat# ab125212, RRID: AB_10975465), anti-nerve growth factor (NGF; rabbit, 1:1000, Abcam, Cat# ab6199, RRID: AB_2152414), and anti-histocompatibility 2, D region locus 1 (H2-D1, rabbit, 1:1000, Bioss, Beijing, China, Cat# bs-15408R, RRID: AB_2348249). The secondary antibodies used were horseradish peroxidase (HRP) goat anti-rabbit IgG (1:5000, ABclonal, Cat# AS029, RRID: AB_2769859) and HRP goat anti-mouse IgG (1:5000, ABclonal, Cat# AS003, RRID: AB_2769851).

### High-throughput RNA sequencing

Total RNA was extracted from brain tissue using TRIzol reagent (Invitrogen, 10296010), according to the manufacturer’s protocol. RNA purity and quantification were determined using a NanoDrop 2000 spectrophotometer (Thermo Fisher Scientific). RNA integrity was assessed using the Agilent 2100 Bioanalyzer (Agilent Technologies, Santa Clara, CA, USA). Libraries were constructed using the TruSeq Stranded mRNA LT Sample Prep Kit (Illumina, San Diego, CA, USA) according to the manufacturer’s instructions. Transcriptome sequencing and analysis including volcano plot, Gene Ontology (GO), and Kyoto Encyclopedia of Genes and Genomes (KEGG) enrichment analyses were conducted by Majorbio Co., Ltd. (Shanghai, China). Libraries were sequenced on an Illumina HiSeq X Ten platform, with 150 bp paired-end reads generated. Raw data (raw reads) in fastq format were first processed using Trimmomatic, with low-quality reads removed to obtain clean reads.

### Statistical analysis

All data are expressed as mean ± standard error of the mean (SEM) and were analyzed using GraphPad Prism 8.3.0 (GraphPad Software, Boston, MA, USA, www.graphpad.com). Student’s *t*-test was used to compare two groups. One-way analysis of variance followed by Dunnett’s *post hoc* test was used for comparisons among three and more groups. *P* < 0.05 was considered statistically significant.

## Results

### Ultrashort wave alleviates behavioral impairments of traumatic brain injury mice

To determine the biological safety of USW treatment on the brain, a daily 7-minute session of USW for seven consecutive days was first performed on normal mouse brain. Hematoxylin–eosin staining of major organs (including brain, heart, liver, spleen, lung, and kidney) found no significant differences between mice treated without or with USW (**Additional Figure 1A**). In addition, routine blood indices (**Additional Figure 1B**) and blood biochemical analysis (**Additional Figure 1C**) also showed no significant changes with USW treatment. These data indicate that USW treatment on the brain for 7 days is safe and compatible.

To determine the therapeutic effect of USW treatment for TBI mice, a series of behavioral tests were performed (**Figure 1A**). On the 3^rd^, 8^th^, 21^st^, and 31^st^ day after injury, the open field test (**Figure 1B**) and elevated plus maze test (**Figure 1C**) reported markedly lower anxiety levels in the TBI + USW group than in the TBI group. In addition to anxiety, the depressive state of mice after USW treatment was also examined. This demonstrated markedly reduced immobility times in both the tail suspension test and forced swimming test in the TBI + USW group compared with the TBI group on the 22^nd^ and 23^rd^ days following injury (**Figure 1D** and **E**).

Aside from neuropsychological defects, TBI also impairs motor and cognitive function (Joy et al., 2019). The rotarod test, Y-maze, and MWM tests were performed to determine the effect of USW therapy following TBI. In the rotarod test, the time, speed, and total distance to fall were markedly higher in the TBI + USW group than in the TBI group (**Figure 1F**). While exploration time and distance in the novel arm of the Y-maze test were markedly higher in the TBI + USW group than in the TBI group (**Figure 1G**). In the MWM test, typical escape routes and escape latency were similar in the USW + TBI group and Sham group (**Figure 1H**). Moreover, the number of escape platform crossings was markedly higher in the TBI + USW group than in the TBI group (**Figure 1H**). Altogether, these indicate that USW treatment can improve the spatial learning impairments and memory ability of TBI mice.

### Ultrashort wave promotes the repair of nerve injury in the brain of traumatic brain injury mice

**Figure 2:**
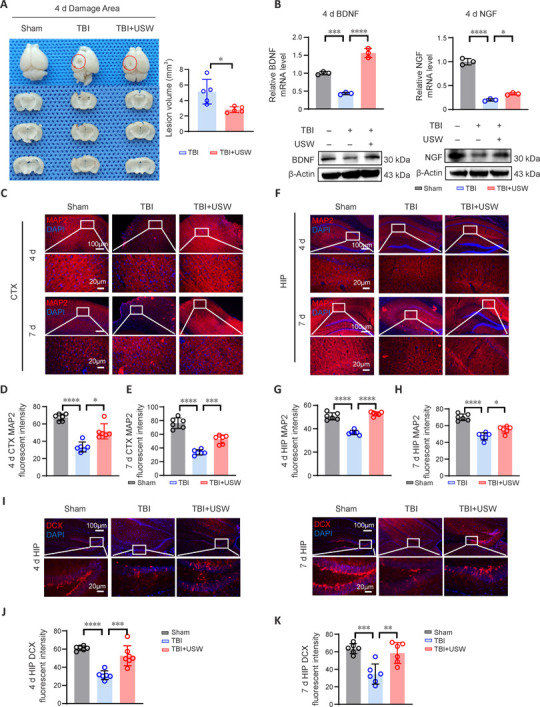
USW repairs neural damage after TBI. (A) USW resulted in a markedly higher level of lesion repair in TBI mice (*n* = 5 mice/group). (B) Real-time PCR and western blotting showed markedly higher levels of BDNF and NGF after USW in TBI mice. (C–H) Representative immunofluorescent images and quantitative analysis of MAP2 (red, Cy3) in the CTX (C–E) and HIP (F–H). Fluorescence intensities were lower in the TBI group compared with the Sham and USW groups. (I–K) Representative immunofluorescent images and quantitative analysis of DCX (red, Cy3) in the HIP. Fluorescence intensity was lower in the TBI group compared with the Sham and USW groups. Scale bars: 100 µm, 20 µm. Data are expressed as mean ± SEM (*n* = 3 mice/group). *P < 0.05, **P < 0.01, ***P < 0.001, ****P < 0.0001 (Student’s *t*-test [A] and one-way analysis of variance followed by Dunnett’s *post hoc* test [B, D, E, G, H, J, K]). BDNF: Brain-derived neurotrophic factor; CTX: cortex; DAPI: 4′,6-diamidino-2–phenylindole; HIP: hippocampus; MAP2: microtubule-associated protein 2; NGF: nerve growth factor; ns: not significant; RT-PCR: real-time polymerase chain reaction; TBI: traumatic brain injury; USW: ultrashort wave.

Neuronal death and diffuse axonal injury are common complications following TBI (Qin et al., 2022). We next explored the effect on brain repair with USW. There was a smaller wound area in the TBI + USW group compared with the TBI group on the 4^th^ day after injury (**Figure 2A**). Next, we examined the expression levels of BDNF and NGF, two neurotrophins crucial for nervous system development, growth, and repair (El-Gazar et al., 2024). Levels of BDNF and NGF were markedly higher in the TBI + USW group than in the TBI group (**Figure 2B**). MAP2 is a neuron-specific protein mainly expressed in axons and dendrites, and is commonly used as a neuronal marker to visualize the morphological characteristics of neurons (Fontanella et al., 2024). On the 4^th^ and 7^th^ days after injury, MAP2 staining in the cortex and hippocampus of the injured area showed that the majority of cortical and hippocampal neurons in the TBI group were broken and disordered, with the intensity of MAP2 staining lower compared with the Sham group (**Figure 2C–H**). In contrast, TBI + USW group mice showed restored neuronal continuity, with higher MAP2 staining intensity than in the TBI group (**Figure 2C–H**). DCX plays an important role in neuronal development, and is mainly involved in the migration and localization of neurons and formation of functional connections (Xie et al., 2024). Similar to MAP2 staining, labeled developing and migrating neurons showed markedly higher fluorescence intensity and complexity of DCX labeling in the hippocampus of the TBI + USW group than in the TBI group (**Figure 2I–K**). Taken together, these results indicate that USW plays a positive role in promoting the recovery of neuronal regeneration in TBI mice.

### Ultrashort wave inhibits glial cell activation and neuroinflammation *in vivo*

**Figure 3:**
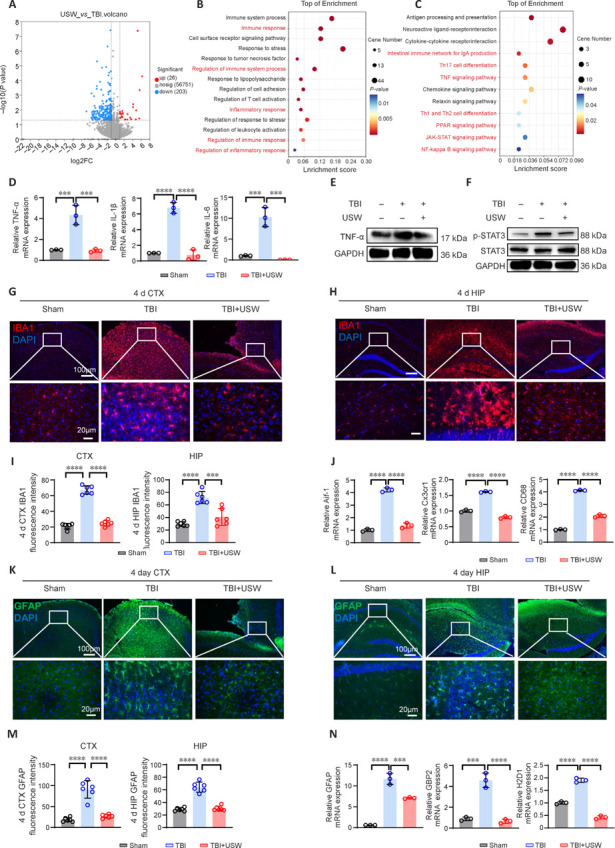
USW reduces pro-inflammatory factor levels and inhibits glial activation after TBI. (A) Transcriptome volcano map of differentially expressed genes between the TBI and TBI + USW groups. (B) GO data showed enrichment of genes in different pathways. (C) KEGG showed enrichment of genes in different pathways. (D) Real-time PCR detected lower mRNA expression of pro-inflammatory factors (TNF-α, IL-1β, and IL-6) in the TBI + USW group than TBI group at 4 days after TBI. (E, F) Western blotting showed lower protein expression of TNF-α (E) and p-STAT3 (F) protein in the TBI + USW group than TBI group at 4 days after TBI. (G–I, K–M) Representative immunofluorescent images of Iba1 staining (red) and GFAP staining (green), with fluorescence intensity (I, M) in the CTX (G, K) and HIP (H, L) at 4 days after TBI. (J, N) Real-time PCR detected lower levels of microglia activation markers (Aif-1, Cx3cr1, and CD68) and astrocyte activation markers (GFAP, GBP2, and H2-D1) in the TBI + USW group than TBI group at 4 days after TBI. Data are expressed as mean ± SEM (*n* = 3 mice/group). ***P < 0.001, ****P < 0.0001 (one-way analysis of variance followed by Dunnett’s *post hoc* test). Scale bars: 100 µm, 20 µm. Aif-1: Allograft inflammatory factor-1; Cx3cr1: C–X3–C motif chemokine receptor 1; DAPI: 4′,6-diamidino-2–phenylindole; GBP2: guanylate-binding protein 2; GFAP: glial fibrillary acidic protein; GO: Gene Ontology; H2-D1: histocompatibility 2, D region locus 1; IL: interleukin; KEGG: Kyoto Encyclopedia of Genes and Genomes; RT-PCR: real-time polymerase chain reaction; TBI: traumatic brain injury; TNF-α: tumor necrosis factor-alpha; USW: ultrashort wave.

To investigate the molecular mechanism of USW therapy, tissue around the brain injury area of mice in the TBI and TBI + USW groups were collected for transcriptome analysis. Volcano plot analysis identified a total of 26 up-regulated and 203 down-regulated genes between the two groups (**Figure 3A**). GO and KEGG enrichment analyses implicated a variety of pathways related to inflammation and immunity (**Figure 3B** and **C**). These results were further supported by markedly decreased mRNA levels of pro-inflammatory factors: TNF-α, IL-1β, and IL-6 (**Figure 3D** and **Additional Figure 2A**); and protein levels of TNF-α and p-STAT3 (**Figure 3E**, **F**, and **Additional Figure 2A–C**) in the TBI + USW group compared with the TBI group. Together, these findings indicate that USW can inhibit neuroinflammation in TBI mice.

Microglia cells are the resident immune cells of the central nervous system and regulate the neuroinflammatory response (Lee et al., 2019). Following TBI, microglia undergo hyper-activation, a phenomenon reflected by up-regulated expression of Iba1 in brain lesions (Schwabenland et al., 2021). On the 4^th^ and 7^th^ day following injury, we observed increased Iba1 staining in the cortex (**Figure 3G–I** and **Additional Figure 2D–F**) and hippocampus (**Figure 3H**, **I**, and **Additional Figure 2E**, **F**) of TBI mice compared with Sham mice. In contrast, mice in the TBI + USW group exhibited markedly reduced staining compared to TBI mice (**Figure 3G–I**, and **Additional Figure 2D–F**), suggesting that USW effectively reduced microglial activation. Additionally, expression of allograft inflammatory factor-1 (Aif-1), C-X3-C motif chemokine receptor 1 (Cx3cr1), and CD68 (**Figure 3J** and **Additional Figure 2G**) were markedly suppressed in the TBI + USW group compared with the TBI group. All three proteins being indicative of microglia activation.

Brain injury is usually accompanied by increased reactive astrocytes (Cameron et al., 2024). Therefore, we used GFAP to label astrocytes in brain tissue of the three groups. On the 4^th^ and 7^th^ day, fluorescence intensities of GFAP immunostaining in the cortex (**Figure 3K**, **M** and **Additional Figure 2H**, **J**) and hippocampus (**Figure 3L**, **M** and **Additional Figure 2I**, **J**) were markedly lower in the TBI + USW group compared with the TBI group, but comparable to the Sham group. Furthermore, mRNA levels of GFAP, GBP2, and H2-D1 reflect astrocyte activation, but were dramatically inhibited in the TBI + USW group compared with the TBI group (**Figure 3N** and **Additional Figure 2H–K**).

Collectively, these results demonstrate that USW markedly suppressed neuroinflammation and microglia and astrocyte activation in TBI mice.

### Ultrashort wave reduces glial cell activation *in vitro*

**Figure 4:**
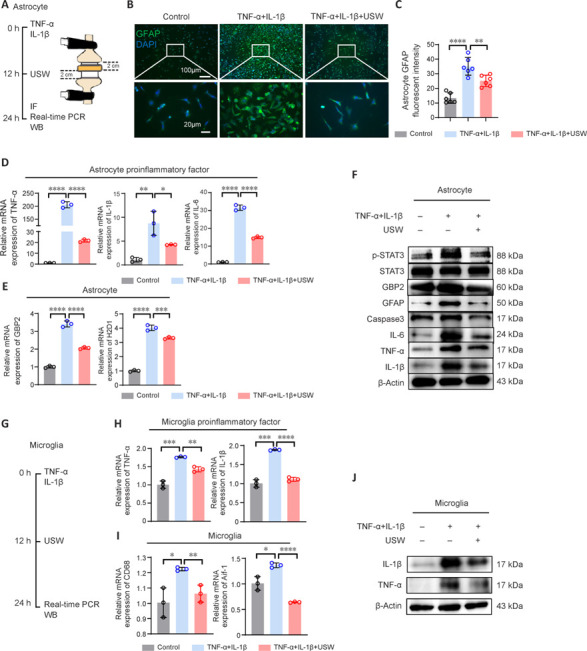
USW reduces levels of inflammatory factors and inhibits activation of astrocytes and microglia *in vitro*. (A) Schematic representation of astrocyte processing. (B, C) Representative immunofluorescent images and quantitative analysis of GFAP (green, Alexa 488). Fluorescence intensity was higher in the TNF-α + IL-1β group compared with the Control and TNF-α + IL-1β + USW groups. Scale bars: 100 µm, 20 µm. (D, E) Real-time PCR showed that USW decreased mRNA expression of pro-inflammatory factors (TNF-α, IL-1β, and IL-6) and astrocyte activation markers (GBP2 and H2-D1) in activated astrocytes. (F) Western blotting showed that USW lowered the protein expression of p-STAT3, GFAP, GBP2, caspase-3, IL-6, IL-1β, and TNF-α in activated astrocytes. (G) Schematic representation of microglia processing. (H, I) RT-PCR showed that USW lowered the mRNA expression of pro-inflammatory factors (TNF-α, and IL-1β) and microglia activation markers (CD68 and Aif-1) in activated microglia. (J) Western blotting showed that USW lowered IL-1β and TNF-α protein expression in activated microglia. Data are expressed as mean ± SEM (*n* = 3/group). *P < 0.05, **P < 0.01, ***P < 0.001, ****P < 0.0001 (one-way analysis of variance followed by Dunnett’s *post hoc* test). Aif-1: Allograft inflammatory factor-1; DAPI: 4′,6-diamidino-2–phenylindole; GBP2: guanylate-binding protein 2; GFAP: glial fibrillary acidic protein; H2-D1: histocompatibility 2, D region locus 1; IF: immunofluorescence; IL: interleukin; p-STAT3: phosphorylated signal transducer and activator of transcription 3; RT-PCR: real-time polymerase chain reaction; STAT3: signal transducer and activator of transcription 3; TNF-α: tumor necrosis factor-alpha; USW: ultrashort wave; WB: Western blot.

To further investigate the effect of USW on reducing the levels of pro-inflammatory factors and inhibiting astrocyte and microglia activation, we performed *in vitro* cell-based experiments. An astrocyte cell line, C8-D1A, was stimulated with TNF-α + IL-1β for 24 hours, with USW performed after stimulation (**Figure 4A**). As shown in **Figure 4B** and **C**, immunofluorescence staining and GFAP intensity were much lower in the TNF-α + IL-1β + USW group compared with the TNF-α + IL-1β group. In addition, real-time PCR showed markedly suppressed expression of inflammatory factors, such as TNF-α, IL-1β, and IL-6 (**Figure 4D**), and markers for astrocyte activation, GBP2 and H2-D1 (**Figure 4E**), after USW treatment. Furthermore, western blotting showed much lower expression of p-STAT3, GFAP, GBP2, caspase-3, IL-6, IL-1β, and TNF-α in the TNF-α + IL-1β + USW group compared with the TNF-α + IL-1β group (**Figure 4F**). To examine the effect of USW on microglial cells, BV2 cells underwent the same treatment as C8-D1A cells (**Figure 4G**). Real-time PCR showed that TNF-α + IL-1β stimulated TNF-α, IL-1β, CD68, and Aif-1 expression in BV2 cells, while USW markedly inhibited their expression (**Figure 4H** and **I**). Western blotting showed that expression of IL-1β and TNF-α were much lower in the TNF-α + IL-1β + USW group compared with the TNF-α + IL-1β group (**Figure 4J**). These data demonstrate that USW suppressed the activation of astrocytes and microglia and limited pro-inflammatory factor levels *in vitro*.

### Ultrashort wave suppresses high Piezo1 protein expression in traumatic brain injury mice

**Figure 5:**
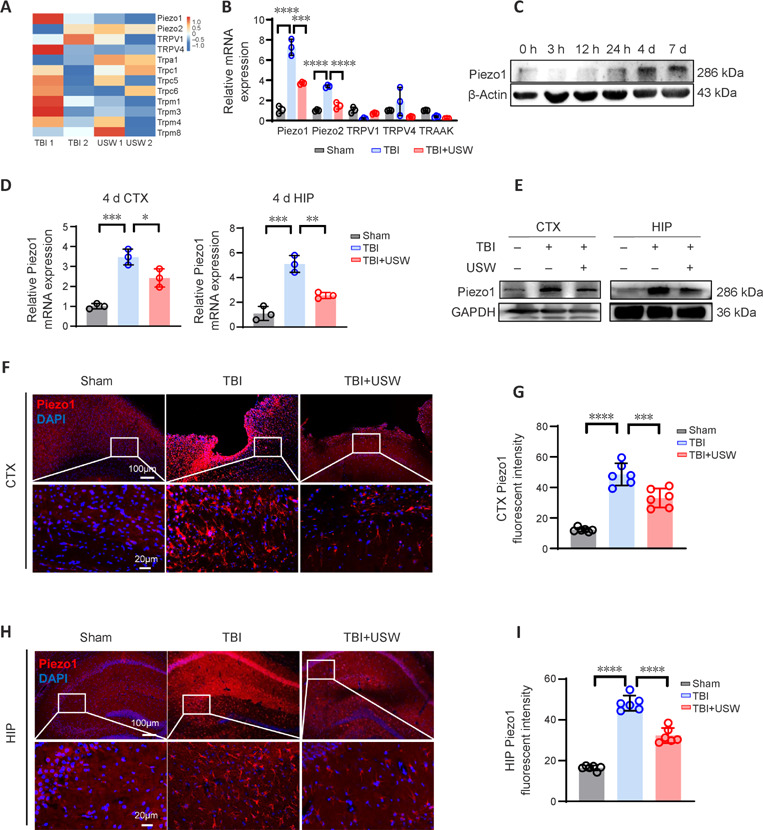
USW reduces high expression of Piezo1 protein after TBI. (A) Transcriptomic heatmap of genes involved in mechanical sensitivity. (B) Real-time PCR detected highest levels of Piezo1 compared with the other genes after TBI. (C) Western blotting showing temporal expression of Piezo1 protein after TBI. (D–I) Real-time PCR (D), western blotting (E), and immunofluorescent staining (red, Cy3) (F–I) showed lower levels of Piezo1 in the TBI + USW group than TBI group. Scale bars: 100 µm, 20 µm. Data are expressed as mean ± SEM (*n* = 3 mice/group). *P < 0.05, **P < 0.01, ***P < 0.001, ****P < 0.0001 (one-way analysis of variance followed by Dunnett’s *post hoc* test). CTX: Cortex; DAPI: 4′,6-diamidino-2–phenylindole; Gapdh: glyceraldehyde-3-phosphate dehydrogenase; HIP: hippocampus; RT-PCR: real-time polymerase chain reaction; TBI: traumatic brain injury; TRAAK: potassium channel subfamily K member 4; TRPV1: transient receptor potential vanilloid 1; TRPV4: transient receptor potential vanilloid 4; USW: ultrashort wave.

The principle of USW is based on the capacitive field method, whereby a mechanical force is applied to the microscopic surface, which alters cell surface tension (Aydin and Akar, 2011; Bouji et al., 2020; Tenenbaum, 2024). Given the importance of mechanosensitive calcium ion channels in mediating cellular responses to physical stimuli, we performed transcriptome sequencing on brain tissue from TBI and TBI + USW group mice on the 4^th^ day following injury. We examined mechanical sensitivity genes including Piezo1 (Yang et al., 2022a), Piezo2 (Ma et al., 2023), transient receptor potential vanilloid 1 (TRPV1; Luo et al., 2021), transient receptor potential vanilloid 4 (TRPV4; Di et al., 2023), and potassium channel subfamily K member 4 (TRAAK; (Idoux et al., 2023). Down-regulation of these genes was observed in the TBI + USW group (**Figure 5A**). Notably, Piezo1 increased most dramatically in TBI mice, while USW markedly inhibited its expression (**Figure 5A** and **B**). This implies that Piezo1 might play an important role in TBI. To test this hypothesis, we examined a time gradient of Piezo1 expression after TBI. Western blotting showed that Piezo1 protein expression gradually increased after TBI, peaking at day 4 (**Figure 5C**). Moreover, real-time PCR, western blotting, and immunofluorescence staining showed that Piezo1 expression was higher in the cortex and hippocampus of the TBI group on the 4^th^ day after injury, compared with the Sham group. Meanwhile USW markedly inhibited Piezo1 expression (**Figure 5D–I**). These results indicate that USW reduced Piezo1 overexpression in TBI mice.

### Ultrashort wave alleviates behavioral impairments by suppressing Piezo1 expression

**Figure 6:**
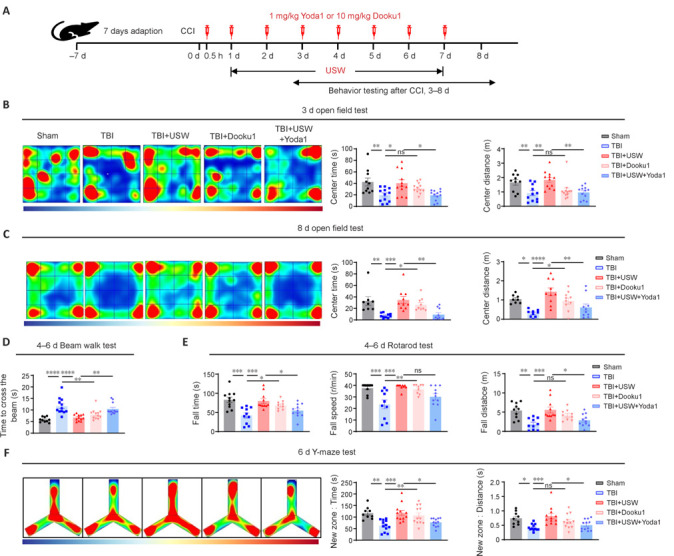
USW ameliorates nerve damage after TBI by reducing Piezo1 protein levels. (A) Schematic diagram of mouse treatment strategy. (B, C) Mice in the TBI + Dooku1 group had lower levels of anxiety compared with the TBI group. Mice in the TBI + USW + Yoda1 group had higher levels of anxiety compared with the TBI + USW group, measured by the open field test on the 3^rd^ and 8^th^ days. (D, E) Mice in the TBI + Dooku1 group had a higher level of motor ability compared with the TBI group. Mice in the TBI + USW + Yoda1 group had a lower level of motor ability compared with the TBI + USW group. Motor ability was measured by beam walk test (D) and rotarod test (E). (F) Mice in the TBI + Dooku1 group had higher levels of cognitive ability compared with the TBI group. Mice in the TBI + USW + Yoda1 group had lower levels of cognitive ability compared with the TBI + USW group. Cognitive ability was measured using the Y-maze test. Data are expressed as mean ± SEM (*n* = 10 [Sham] or 12 [TBI, TBI + USW, TBI + Dooku1, and TBI + USW + Yoda1]). *P < 0.05, **P < 0.01, ***P < 0.001, ****P < 0.0001 (one-way analysis of variance followed by Dunnett’s *post hoc* test). CCI: Controlled cortical impact; Dooku1: Piezo1 inhibitor; ns: not significant; TBI: traumatic brain injury; USW: ultrashort wave; Yoda1: Piezo1 agonist.

To investigate the effects of USW on Piezo1 following TBI, we performed behavioral tests after injecting the Piezo1 agonist, Yoda1, to TBI + USW mice, or its inhibitor, Dooku1, to TBI mice (**Figure 6A**). On the 3rd and 8th day, mice in both the TBI + Dooku1 and TBI + USW groups demonstrated a relief of anxiety behaviors in the open field test, compared to the TBI group (**Figure 6B** and **C**). Yoda1 injection reversed these improved effects of USW on TBI mice, with less center time and center distances observed in the TBI + USW + Yoda1 group compared with the TBI + USW group (**Figure 6B** and **C**). Interestingly, in the balance beam test (which evaluates the exercise capabilities and balance of mice), mice in both the TBI + USW and TBI + Dooku1 groups took less time to cross the beam compared with the TBI group (**Figure 6D**). Similarly, in the rotarod test and Y-maze test, the TBI + USW group and TBI + Dooku1 group demonstrated improved behaviors (**Figure 6E** and **F**). However, Yoda1 injection reversed the USW therapeutic effect on TBI mice in the balance beam test, rotarod test, and Y-maze test (**Figure 6D–F**). Altogether, these data show that USW improved behavioral impairments of TBI mice by reducing Piezo1 protein expression.

### Ultrashort wave inhibits astrocyte activation by suppressing Piezo1 expression

**Figure 7:**
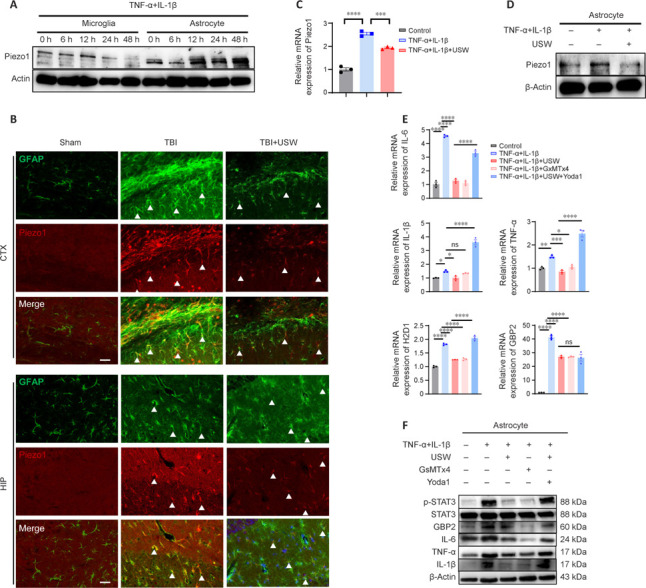
USW reduces pro-inflammatory factor levels by decreasing Piezo1 protein in astrocytes. (A) Western blotting showing temporal expression of Piezo1 protein in C8-D1A astrocytes and BV2 microglia under the same inflammatory conditions. (B) Representative immunofluorescent images of GFAP (green, Alexa 488) and Piezo1 (red, Cy3). The number of GFAP-positive cells and Piezo1-positive cells was higher in the TBI group than the Sham group and USW group. Arrows represent co-localized cells. Scale bars: 20 µm. (C, D) Real-time PCR (C) and western blotting (D) detected lower levels of Piezo1 in the TNF-α + IL-1β + USW group compared with the TNF-α + IL-1β group. (E, F) The TNF-α + IL-1β + GsMTx4 group had lower levels of pro-inflammatory proteins (TNF-α, IL-1β, IL-6, and p-STAT3) and astrocyte activation markers (GBP2 and H2-D1) compared with the TNF-α + IL-1β group. The TNF-α + IL-1β + USW + Yoda1 group showed reversal of the anti-inflammatory effects of USW. Data are expressed as mean ± SEM (*n* = 3/group). *P < 0.05, **P < 0.01, ***P < 0.001, ****P < 0.0001 (one-way analysis of variance followed by Dunnett’s *post hoc* test). GBP2: Guanylate-binding protein 2; GFAP: glial fibrillary acidic protein; H2-D1: histocompatibility 2, D region locus 1; IL: interleukin; ns: not significant; p-STAT3: phosphorylated signal transducer and activator of transcription 3; TBI: traumatic brain injury; TNF-α: tumor necrosis factor-alpha; USW: ultrashort wave; Yoda1: Piezo1 agonist.

To determine what cells are impacted by USW reduction in Piezo1 protein after TBI, we focused on astrocytes and microglia, as these cell types are widely involved in brain tissue inflammation. Western blotting showed that under inflammatory activation conditions, Piezo1 expression gradually increased in astrocytes and was much higher compared with microglia after 12 hours of TNF-α + IL-1β stimulation (**Figure 7A**). We then mainly studied the relationship between astrocytes and Piezo1 in the brain of TBI mice. Immunofluorescence staining in the cortex and hippocampus confirmed that Piezo1 expression colocalized with the astrocyte marker, GFAP. USW treatment reduced the expression of both Piezo1 and GFAP (**Figure 7B**).

Next, the effect of USW on Piezo1 expression in C8-D1A astrocytes *in vitro* was tested. Considering that Piezo1 expression peaked at 12 hours after stimulation (**Figure 7A**), a single USW treatment was applied at 12 hours, and the cells were collected at 24 hours of TNF-α + IL-1β stimulation. Real-time PCR and western blotting showed that Piezo1 expression was markedly higher in the TNF-α + IL-1β group compared with the Control group, while USW reduced high Piezo1 expression in C8-D1A astrocytes (**Figure 7C** and **D**).

To further investigate the function of Piezo1 on the anti-inflammatory effects of USW, we added the Piezo1 agonist, Yoda1, or inhibitor, GsMTx4, to the astrocyte inflammatory activation model. Both GBP2 and H2-D1 are associated with activation of astrocytes, and their expression levels are positively correlated with pro-inflammatory polarization of astrocytes (Weiss et al., 2024; Sun et al., 2025). Real-time PCR showed that GsMTx4 addition and USW treatment, dramatically inhibited high expression of TNF-α, IL-1β, IL-6, GBP2, and H2-D1 after TNF-α + IL-1β stimulation (**Figure 7E**). Interestingly, Yoda1 addition reversed the suppressive effect of USW treatment on the expression of pro-inflammatory factors and astrocyte activation markers (**Figure 7E**). Accordingly, western blotting revealed that similar to USW, GsMTx4 markedly inhibited expression of p-STAT3, GBP2, IL-1β, IL-6, and TNF-α, while Yoda1 reversed this suppressive effect (**Figure 7F**). Altogether, these data demonstrate that USW inhibited astrocyte activation and pro-inflammatory factor production by suppressing Piezo1 expression.

## Discussion

In this study, we show that USW can facilitate injury repair and alleviate emotional and cognitive disorders in TBI mice. Furthermore, USW inhibited neuroinflammation and promoted neuronal regeneration. Notably, USW down-regulated Piezo1 protein expression in astrocytes, thereby further promoting injury repair and nerve recovery post-TBI.

Neuroinflammation and immune responses triggered following TBI (including cytokine release and production of inflammatory mediators) give rise to neuronal damage and cause cognitive and behavioral dysfunction (Yang et al., 2023). Moreover, long-term neuroinflammation increases the risk of developing neurodegenerative diseases such as Alzheimer’s disease and Parkinson’s disease (Abou-El-Hassan et al., 2023; Billiar et al., 2023; Ravichandran and Heneka, 2024). Therefore, controlling and mitigating neuroinflammation after TBI is essential to promote recovery and restore brain function. Anti-inflammatory drugs such as the steroid dexamethasone (Jones et al., 2023), simvastatin (Potey et al., 2015; Rodríguez-Perea et al., 2017), and atorvastatin (Xu et al., 2017) have shown rapid effects in alleviating brain damage after TBI. However, their side effects cannot be ignored and include immune system suppression. Compared with drug treatments and stem cell treatments (Qin et al., 2022), USW also has a rapid inhibitory effect on neuroinflammation. In this study, we found that USW could rapidly inhibit neuroinflammation on the 4th day after TBI, reduce the activation of astrocytes and microglia, promote nerve regeneration, and alleviate behavioral deficits of TBI mice. Furthermore, USW has additional advantages of being painless, non-invasive, a non-drug, and highly safe. It has been widely used for the clinical treatment of rhinitis, tonsillitis, otitis media, and ocular inflammation (Xu et al., 2021; Wu et al., 2022). Some studies have combined USW with human umbilical cord mesenchymal stem cells to treat spinal cord injury, showing that USW plays a more important role than human umbilical cord mesenchymal stem cells alone in terms of reducing neuroinflammation (Na et al., 2020). This is consistent with our study showing that USW can inhibit neuroinflammation and improve neurological impairment. Our previous studies have shown that adhesive and conductive hydrogels and ectoderm-derived frontal bone mesenchymal stem cells (Qin et al., 2022; Yan et al., 2023) can suppress neuroinflammation and efficiently promote TBI. However, comparing the time-consuming and costly processes for hydrogels, stem cells, and drug preparations, USW emerges as a more convenient and economical strategy for TBI therapy.

Piezo1 is tightly associated with inflammation. In the context of atherogenesis, oxidized low-density lipoprotein causes up-regulation of Piezo1 expression in endothelial cells. This subsequently leads to increased Yes-associated protein nuclear translocation and increased levels of c-Jun N-terminal kinase, nuclear factor-κB, and TNF-α (Yang et al., 2022c). Alternatively, inhibiting the Piezo1 channel by siRNA targeting Piezo1 or the inhibitor GsMTx4 can alleviate these adverse effect (Yang et al., 2022c). The Piezo1 channel may be involved in neuroinflammation characterized by reactive astrocytes. Velasco-Estevez et al. (2020) have shown that lipopolysaccharide-induced neuroinflammation can increase Piezo1 expression in astrocytes. In our study, we show that TBI induced elevated expression of Piezo1, while inhibiting Piezo1 with Piezo1 inhibitor GsMTx4 or Dooku1 promoted neurological dysfunction recovery and reduced inflammatory factor levels. Activating Piezo1 with the agonist Yoda1 reversed these beneficial effects of USW on brain injury and astrocyte activation, confirming the suppressive function of USW on Piezo1.

USW is a high-frequency electric field treatment, which can alter cell membrane tension (Aydin and Akar, 2011; Bouji et al., 2020; Tenenbaum, 2024). Although Piezo1 is a membrane protein for mechanical sensitivity, USW may indirectly modulate Piezo1 function through action on the cell membrane. Physical factor therapies similar to USW, such as ultrasound and transcranial magneto–acoustic stimulation, also regulate expression of Piezo1 protein (Chu et al., 2023; Zhang et al., 2023). A previous study demonstrated that ultrasound is capable of inhibiting high Piezo1 expression in spinal cord injury mice (Zhang et al., 2023). Transcranial magneto–acoustic stimulation can activate microglial Piezo1 to alleviate neuroinflammation, synaptic plasticity impairment, and neural oscillation abnormalities in 5×FAD mice (Chu et al., 2023). Compared with these physical factor therapies, USW has the advantages of being highly penetrant and rapid. Our results show that USW can inhibit Piezo1 expression. Whether USW might open or close Piezo1 channels and then regulate the influx of calcium ions requires further investigation.

Transcranial magnetic stimulation and transcranial direct current stimulation are non-invasive physical therapies similar to USW. Transcranial magnetic stimulation is a non-invasive neuromodulation technique that employs magnetic fields to stimulate nerve cells in the brain (Castel-Lacanal et al., 2014). Transcranial magnetic stimulation is capable of suppressing neuroinflammation after TBI (Qian et al., 2024). Transcranial direct current stimulation is a non-invasive brain stimulation technique that involves passing a low voltage electrical current across the scalp (Chase et al., 2020). Transcranial direct current stimulation can improve balance function (Park et al., 2021) and neural plasticity (Kim and Han, 2017) in the rat model of repeated mild TBI. Compared with these approaches, USW has a high frequency and relatively deep penetration depth. Therefore, it can effectively penetrate the skull and reach deeper regions inside the brain. Under correct operation, USW has relatively minor side effects and mainly exerts its therapeutic effects through non-thermal effects generated by the high-frequency electric field. Specific frequencies of 27.12 and 40.68 MHz are widely used for the treatment of various diseases. However, to date, no studies have directly compared the therapeutic benefits of these two frequencies. Conducting comparative research to determine the effects and underlying mechanisms of 27.12 and 40.68 MHz in treating TBI or other brain disorders would be highly beneficial. Such comparisons would provide valuable insights and potentially enhance the application of USW therapy across a broader range of conditions.

Our research has found that USW can promote injury repair and nerve regeneration after TBI. USW can promote the secretion of BDNF and NGF, which likely facilitates nerve regeneration. In addition, USW also inhibits astrocyte activation by reducing Piezo1 expression and neuroinflammation, thus maintaining a favorable microenvironment to promote nerve regeneration. In previous studies, USW promoted the repair of peripheral nerve injuries in rabbits (Zhu et al., 2020), which is consistent with our results, indicating that USW has a certain promotive effect on nerve injury repair.

Our study has several limitations. First, our focus was primarily on the effects of USW therapy on Piezo1 expression in astrocytes. However, Piezo1 is also expressed in neurons, microglia, and other cell types. Therefore, further investigation into the role of Piezo1 in these cell types is needed to fully explore the beneficial effects of USW on TBI. Second, we only used Piezo1 inhibitors to examine the function of Piezo1. While this approach provided valuable insights, the use of cell type-specific Piezo1 knockout mice in future studies would more definitively clarify the effects of Piezo1 in different cellular contexts.

Overall, our study shows that USW treatment can efficiently promote brain injury recovery, and that USW may be an ideal therapeutic strategy for TBI. Furthermore, Piezo1 can be a potential therapeutic target for TBI and other neurological disorders involving neuroinflammation.

## Additional files:

***Additional Figure 1:*** *Validation of USW biosafety.*

Additional Figure 1 Validation of USW biosafety.
(A) Representative images of H&E staining of the brain, heart, liver, spleen, lung, and kidney on the 7th day. There were no differences between the two groups (n = 4 mice/group). Scale bar: 100 μm. (B) Routine blood indices showed no significant differences between control and USW groups, including red blood cell count (RBC), white blood cell count (WBC), hemoglobin (HGB), and platelet count (PLT) (n = 4 mice/group). (C) Blood biochemical indices showed no significant differences between the control group and USW group, including alanine aminotransferase (ALT), aspartate aminotransferase (AST), albumin (ALB), blood urea nitrogen (BUN), serum creatinine (CREA), lactate dehydrogenase (LDH), uric acid (UA), creatine kinase (CK), and urea (n = 9 mice/group). Data are expressed as mean ± SEM and were analyzed by Student’s *t*-test. H&E: Hematoxylin-eosin; ns: no significant; USW: ultrashort wave.

***Additional Figure 2:*** *USW reduces proinflammatory factor levels and inhibits glial activation 7 days after TBI.*

Additional Figure 2 USW reduces pro-inflammatory factor levels and inhibits glial activation 7 days after TBI.
(A) RT-PCR showed lower levels of pro-inflammatory factors (TNF-α, IL-1β, and IL-6) in the TBI + USW group compared with the TBI group. (B) Western blotting showed lower levels of TNF-α (B) and p-STAT3 (C) proteins in the TBI + USW group than TBI group. (D-F, H-J) Representative immunofluorescent images and quantitative analysis of Iba1 (red, Cy3) and GFAP (green, Alexa 488) in the CTX and HIP. Fluorescence intensities of GFAP and Iba1 were higher in the TBI group compared with the Sham and USW groups. (G, K) RT-PCR revealed lower levels of microglia activation markers (Aif-1, Cx3cr1, and CD68) and astrocyte activation markers (GFAP, GBP2, and H2-D1) in the TBI + USW group compared with the TBI group. Data are expressed as mean ± SEM (n = 3 mice/group). ^***^P < 0.001, ^****^P < 0.0001 (one-way analysis of variance followed by Dunnett’s post hoc test). Scale bars: 100 μm, 20 μm. Aif-1: allograft inflammatory factor-1; CTX: cortex; Cx3cr1: C-X3-C motif chemokine receptor 1; DAPI: 4',6-diamidino-2-phenylindole; Gapdh: glyceraldehyde-3-phosphate dehydrogenase; GBP2: guanylate-binding protein 2; GFAP: glial fibrillary acidic protein; H2-D1: histocompatibility 2, D region locus 1; HIP: hippocampus; Iba1: ionized calcium-binding adapter molecule 1; IL: interleukin; p-STAT3: phosphorylated signal transducer and activator of transcription 3; RT-PCR: real-time polymerase chain reaction; TBI: traumatic brain injury; TNF-α: tumor necrosis factor-alpha; USW: ultrashort wave.

***Additional Table 1:*** *Sequences of primers used for real-time polymerase chain reaction analysis.*

## Data Availability

*All data relevant to the study are included in the article or uploaded as Additional files.*
